# Mating to Intact, but Not Vasectomized, Males Elicits Changes in the Endometrial Transcriptome: Insights From the Bovine Model

**DOI:** 10.3389/fcell.2020.00547

**Published:** 2020-07-10

**Authors:** Sandra Recuero, José María Sánchez, Yentel Mateo-Otero, Sandra Bagés-Arnal, Michael McDonald, Susanta K. Behura, Thomas E. Spencer, David A. Kenny, Marc Yeste, Pat Lonergan, Beatriz Fernandez-Fuertes

**Affiliations:** ^1^Biotechnology of Animal and Human Reproduction (TechnoSperm), Department of Biology, Institute of Food and Agricultural Technology, University of Girona, Girona, Spain; ^2^School of Agriculture and Food Science, University College Dublin, Dublin, Ireland; ^3^Division of Animal Sciences, University of Missouri, Columbia, MO, United States; ^4^Animal and Bioscience Research Centre, Teagasc Grange, Meath, Ireland

**Keywords:** seminal plasma, cattle, endometrium, transcriptome, maternal environment

## Abstract

An appropriate female reproductive environment is essential for pregnancy success. In several species, including mice, pigs and horses, seminal plasma (SP) components have been shown to modulate this environment, leading to increased embryo viability and implantation. Due to the characteristics of mating in the aforementioned species, SP comes into direct contact with the uterus. However, it is questionable whether any SP reaches the uterus in species that ejaculate inside the vagina, such as humans and cattle. Hence, we hypothesized that sperm, perhaps acting as a vehicle for SP factors, play a more important role in the modulation of the maternal uterine environment in these species. In addition, changes elicited by SP and/or sperm may originate in the vagina and propagate to more distal regions of the female reproductive tract. To test these hypotheses, a bovine model in which heifers were mated to intact or vasectomized bulls or were left unmated was used. RNA-sequencing of endometrial samples collected 24 h after mating with a vasectomized bull did not reveal any differentially expressed genes (DEGs) in comparison with control samples. However, the endometrium of heifers mated with intact bulls exhibited 24 DEGs when compared to heifers mated with vasectomized bulls, and 22 DEGs when compared to unmated control heifers. The expression of a set of cytokines (*IL6*, *IL1A*, *IL8*, and *TNFA*) and candidate genes identified in the endometrial RNA-sequencing (*PLA2G10*, *CX3CL1*, *C4BPA*, *PRSS2*, *BLA-DQB*, and *CEBPD*) were assessed by RT-qPCR in the vagina and oviductal ampulla. No differences in expression of these genes were observed between treatments in any region. However, mating to both intact and vasectomized bulls induced an increase in *IL1A* and *TNFA* expression in the vagina compared to the oviduct. These data indicate that sperm, but not secretions from the accessory glands alone, induce modest changes in endometrial gene expression after natural mating in cattle. However, it is not clear whether this effect is triggered by inherent sperm proteins or SP proteins bound to sperm surface at the time of ejaculation.

## Introduction

Embryonic loss is a major contributor to pregnancy failure in livestock species and humans, ranging from 20 to 40% (Macklon et al., 2002; Wiltbank et al., 2016). Most of these losses occur before implantation, highlighting the importance of this period that encompasses such critical events as the first embryonic cleavage divisions; embryonic genome activation; blastocyst formation and hatching; conceptus development; and the preparation of the endometrium to interact with the embryonic trophectoderm (Diskin and Morris, 2008; Niakan et al., 2012; Sandra et al., 2017). Many factors are involved in implantation failure, but in mice and pigs there is growing evidence of a role for the maternal immune system and its regulation by seminal plasma (SP) (Gangnuss et al., 2004; O’Leary et al., 2004, 2006; Song et al., 2016; Glynn et al., 2017).

Seminal plasma is a complex fluid resulting from the secretions of the testes, epididymides and accessory sex glands (in the bull: ampullae, seminal vesicles, prostate and bulbourethral glands). Although it is difficult to accurately calculate the precise contribution of each organ and gland to the final composition of this fluid, vasectomy in the bull by removal of a portion of each vas deferens and therefore removing the contribution of the epididymides, does not appear to significantly affect SP volume (Alexander et al., 1971), indicating a more prominent role of the accessory sex glands. However, vasectomy does lead to a reduction in amino acids in the bull ejaculate (Alexander et al., 1971), and to slight modifications in the proteome of human SP (Batruch et al., 2011). Traditionally, SP has been viewed as a mere vehicle for sperm that nourishes and supports these cells in the female reproductive tract. However, mounting evidence demonstrates an emerging role for SP components in the modulation of the endometrial and oviductal environment, which results in improved fertility and embryo survival and development (reviewed in Bromfeld, 2016; Morgan and Watkins, 2020). Exposure to SP in mice (Schjenken et al., 2015; Song et al., 2016; Glynn et al., 2017), as well as in pigs (O’Leary et al., 2004, 2006) and mares (Tunon et al., 2000; Palm et al., 2008; Fedorka et al., 2017), induces the expression of several endometrial cytokines, leading to leukocyte recruitment to the uterus. This migration of immune cells was thought to solely serve the purpose of clearing microorganisms and excess sperm (Pandya and Cohen, 1985; Thompson et al., 1992). However, it is now thought that the endometrial cytokine and chemokine cascade induced by SP is important to facilitate maternal tolerance toward paternal antigens (reviewed in Robertson, 2007). Indeed, in mice, mating drives the expansion of CD4^+^CD25^+^ T regulatory cells (Robertson et al., 2009; Shima et al., 2015), which can suppress or modulate the immune response of other cells (Sakaguchi et al., 2001). The increase in CD4^+^CD25^+^ is not observed when females are mated to vasectomized or seminal-vesicle-excised males, suggesting that this expansion is driven by secretions from the male accessory glands (Robertson et al., 2009). This effect likely explains why mating increases maternal tolerance toward paternal major histocompatibility complex (MHC) antigens (Robertson et al., 2009), which improves the ability of the semi-allogenic embryo to implant and develop normally in this species (Bromfield et al., 2014; Watkins et al., 2018). In addition to modifying the uterine environment, transcervical infusion of SP in pigs has been shown to modulate ovarian function by increasing corpora lutea (CL) weight and progesterone synthesis (O’Leary et al., 2006), which is essential for creating an appropriate uterine environment for the developing embryo. Moreover, in horses, a pivotal role of SP in protecting spermatozoa from neutrophil phagocytosis in the uterus has been suggested, improving fertility in this environment (Troedsson et al., 2002; Alghamdi et al., 2004).

Due to characteristics of mating in rodents, pigs and horses, SP reaches the uterus and can therefore interact directly with the endometrium (Hunter, 1981; Dean et al., 2011). It is not clear, however, whether any SP reaches the uterus in species that ejaculate intravaginally and in which the volume of the ejaculate is relatively low, such as cattle or humans. It is possible that in those species, SP has an indirect effect on the endometrial environment and/or that sperm act as vehicles for the transport of SP components to more distal regions of the reproductive tract. In this sense, the bovine model could be more appropriate than rodents or pigs in understanding the regulatory properties of SP in the maternal environment of women.

*In vitro* studies in humans have demonstrated the potential of SP to induce expression of cytokines and chemokines in vaginal, cervical and endometrial epithelial cell cultures (Gutsche et al., 2003; Sharkey et al., 2007, 2012a; Remes Lenicov et al., 2012). While gene expression changes and leukocyte recruitment have been described in the human cervix after unprotected, but not condom-protected, coitus (Sharkey et al., 2012b), there is currently no evidence of SP-induced changes in the endometrium *in vivo*.

In cattle, the expression of several inflammatory mediators (such as colony-stimulating factor 2 – *CSF2*, interleukins 1B, 6, 17A and 8 - *IL1B*, *IL6*, *IL17A*, *IL8*; Prostaglandin-endoperoxide synthase 2 – *PTGS2*, and transforming growth factor beta 1 – *TGF-B1*) in uterine horns ipsi- and contralateral to the CL was modified after SP infusion into the uterus, in the absence or presence of sperm (Ibrahim et al., 2019). Despite this, uterine infusion of SP at the time of artificial insemination (AI) does not increase pregnancy rate in heifers or cows (Odhiambo et al., 2009; Ortiz et al., 2019). As mentioned above, it is questionable whether SP reaches the uterus during mating in cattle, so results obtained from the infusion of SP into the uterus may not be representative of physiological conditions. Indeed, recently it has been shown that infusion of SP into the vagina, but not into the uterus, modifies endometrial levels of epidermal growth factor (Badrakh et al., 2020), which highlights the importance of considering the ejaculate deposition site in natural conception in these studies. We recently reported a modest increase in conceptus length in embryos that developed from Day 7 to Day 14 in the uterus of heifers mated to a vasectomized bull in comparison to unmated heifers (Mateo-Otero et al., 2020). However, exposure of heifers to vasectomized bulls prior to AI failed to increase pregnancy rates (Pfeiffer et al., 2012). In addition, although bulls that had their seminal vesicles resected exhibited reduced semen volume, there was no apparent effect on their subsequent fertility (Shah et al., 1968). Together with recent work from our group, demonstrating a deleterious effect of bull SP on endometrial RNA integrity due to the presence of a seminal RNase (Fernandez-fuertes et al., 2019), the literature seems to suggest that SP does not play a significant role in pregnancy establishment in cattle.

Based on these data, we hypothesized that in species that ejaculate inside the vagina, changes in the female reproductive environment begin in this region and then propagate to more distal regions, such as the uterus and/or the oviduct. Also, because of the lack of direct contact with the seminal fluid, sperm probably play a more important role in the modulation of the uterine environment in these species. In order to test these hypotheses, RNA-sequencing analysis of endometrial samples was carried out following natural mating of heifers with vasectomized (whose ejaculate lack sperm and epididymal and testicular fluid) or intact (that ejaculate sperm and SP) bulls. In addition, the expression of a set of interesting candidate genes was assessed in the vagina and oviductal ampulla, with the aim of determining the effects of sperm and accessory gland secretions from the most proximal region of the female reproductive tract (vagina) to the distal region where gamete interaction takes place (oviductal ampulla).

## Materials and Methods

Unless otherwise stated, all chemicals and reagents were sourced from Sigma-Aldrich (Arklow, Ireland).

### Animals

All experimental procedures involving animals were approved by the Animal Research Ethics Committee of University College Dublin and licensed by the Health Products Regulatory Authority (HPRA), Ireland, in accordance with Statutory Instrument No. 543 of 2012 (under Directive 2010/63/EU on the Protection of Animals used for Scientific Purposes). For the duration of the study, all animals were housed in groups of 10–15, independent of treatment, and managed identically in terms of feeding and husbandry routines.

Vasectomy was carried out by removing approximately 5 cm of both vasa deferentia. This procedure took place 5–6 months prior to the trial. Vasectomized bulls underwent semen evaluation to confirm the lack of sperm and all ran as teasers for oestrus detection with 25 cows/heifers each during the breeding season preceding the study. Intact bulls underwent a breeding soundness evaluation prior to the study. Neither intact and vasectomized bulls had access to females for at least 5 months before the trial, nor during it (apart from the controlled mating to the experimental heifers).

### Experimental Design

**FIGURE 1:**
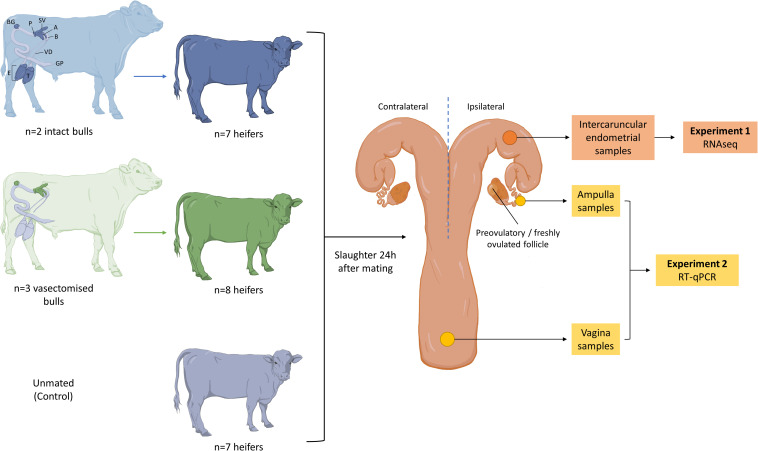
Summary of the experimental design. Only heifers observed in standing estrus were mated with (1) an intact bull (blue; *n* = 7), (2) a vasectomized bull (green; *n* = 8) or (3) left unmated (gray; *n* = 7). A schematic representation of the male reproductive tract is shown, and the structures that contribute to the ejaculate in each case are highlighted (T, testis; E, epididymis; GP, glans penis; VD, vas deferens; B, bladder; A, ampulla; SV, seminal vesicle; P, prostate; BG, bulbourethral gland). Heifers were slaughtered 24 h after mating and their reproductive tracts were recovered. Endometrial samples were obtained from the base of the ipsilateral uterine horn to perform RNA sequencing (Experiment 1). Samples from ampulla and vagina were obtained to assess the expression of a set of genes (*PLA2G10*, *CX3CL1*, *C4BPA*, *PRSS2*, *BLA-DQB*, *CEBPD*, *IL1A*, *IL6*, *TNFA*, and *IL8*) by RT-qPCR (Experiment 2).

Estrous cycles of crossbreed beef heifers (Angus and Holstein-Friesian cross; *n* = 28) were synchronized using an 8-day intravaginal device (PRID^®^ Delta, 1.55 g progesterone, Ceva Santé Animale, Libourne, France), together with a 2 mL intramuscular injection of a synthetic gonadotrophin releasing hormone (Ovarelin^®^, equivalent to 100 μg Gonadorelin, Ceva Santé Animale) administered on the day of PRID insertion. One day prior to PRID removal, all heifers received a 5 mL intramuscular injection of prostaglandin F2 alpha (Enzaprost^®^, equivalent to 25 mg of Dinoprost, Ceva Santé Animale) to induce luteolysis. Only heifers observed in standing estrus were used (*n* = 22). Heifers were blocked by weight and randomly allocated to one of three treatments (Figure 1): (1) mated to an intact bull (*n* = 7), (2) mated to a vasectomized bull (*n* = 8), or (3) left unmated (control; *n* = 7). Between 0 to 6 h after estrus detection, heifers were separated from the group and placed in a pen (one at a time) with one of three vasectomized Holstein Friesian bulls, or one of two intact Holstein Friesian bulls (Supplementary Table 1). Once the bull mounted and intromission was confirmed, the heifers were returned to the group. Bulls were allowed to mate no more than twice per day and the experiment was carried out over three consecutive days.

### Tissue Collection

All heifers were slaughtered in a commercial abattoir 24 h (±6 h) after mating and their reproductive tracts were recovered. The ovaries were examined to determine the site of the preovulatory or freshly ovulated follicle (Supplementary Figure 1). Endometrial tissue samples were obtained from intercaruncular areas of the base of uterine horn ipsilateral to the preovulatory or freshly ovulated follicle. In cattle, the uterine glands are located in the intercaruncular areas of the endometrium, while the caruncular areas are aglandular. These glands are responsible for the secretion of the histotroph, which will nurture the developing embryo, and drive the maternal signals of implantation (Kelleher et al., 2019; Spencer et al., 2019). In addition, epithelial sections of anterior vagina and whole sections of the ampulla of the ipsilateral oviduct were obtained. Immediately after tissue collection, each sample was snap frozen in liquid nitrogen and stored at −80°C.

### Experiment 1: Seminal Plasma Effects on the Endometrial Transcriptome

#### RNA Extraction

For total mRNA extraction, samples were first homogenized in Trizol reagent (Invitrogen, Carlsbad, CA, United States) using a steel bead and the Qiagen tissue lyzer (2 × 120 s at maximum speed). On-column RNA purification was performed using the Qiagen RNeasy kit (Qiagen, Crawley, Sussex, United Kingdom) per the manufacturer’s instructions. The quantity of RNA was determined using the Nano Drop 1000 spectrophotometer (Thermo Fisher Scientific, Dublin, Ireland). Prior to endometrial RNA sequencing analysis, the RNA quality was assessed by the Agilent Bioanalyzer (Agilent Technologies, Cork, Ireland). Only samples that exhibited a minimum RNA integrity number (RIN) of 8 were used in this experiment (*n* = 6 heifers in each experimental group).

#### RNA Sequencing Analysis

RNA library preparation and sequencing were performed by the University of Missouri DNA Core Facility as described previously by Moraes et al. (2018). The raw sequences (fastq) were subjected to quality trimming control using fqtrim^1^. Then, the quality reads were mapped to the bovine reference genome UMD3.1 using Hisat2 mapper (Kim et al., 2015). Read counts mapping to each gene were determined from the binary alignment map files of the samples using FeatureCounts (Liao et al., 2014). Differential expression analysis between different sample groups was performed using edgeR robust (Zhou et al., 2014).

For the annotated DEGs, the gene ontology analysis was performed using PANTHER^2^.

### Experiment 2: Seminal Plasma Effects on Gene Expression in the Vagina and Oviduct

#### RNA Extraction and cDNA Synthesis

Total mRNA extraction was carried out as described above. For each sample, cDNA was prepared from approximately 100 ng of total mRNA using the High Capacity cDNA Reverse Transcription Kit (Thermo Fisher Scientific) according to the manufacturer’s instructions. The cDNA obtained was diluted using RNase- and DNase-free water in a final concentration of 5 ng/mL and in a total volume of 100 μL. The diluted cDNA samples were stored at −20°C for subsequent analysis. As some samples were lost during the management of the experiment, the number of samples per group varies for the vagina region: *n* = 6 heifers in the control group, *n* = 5 in the intact group and *n* = 7 in the vasectomized group. For the ampulla region, there were *n* = 7 individuals in each experimental group (control, intact, and vasectomized). In addition, the RNA quality was assessed by the Agilent Bioanalyzer (Agilent Technologies, Cork, Ireland) (RIN = 7.81 ± 0.29; mean ± standard error of the mean, SEM).

#### Genes of Interest Selection for Expression Analysis

In order to determine SP effects on gene expression in vagina and oviduct regions, a set of genes (*IL1A*, *IL6*, *TNFA*, and *IL8*) were selected based on the literature. These genes were inflammatory mediators expression of which was reported to be modified by SP exposure in cattle and other species (O’Leary et al., 2004; Sharkey et al., 2012b; Schjenken et al., 2015; Introini et al., 2017; Ibrahim et al., 2019). Moreover, expression of some genes (*PLA2G10*, *CX3CL1*, *C4BPA*, *PRSS2*, *BLA-DQB*, and *CEBPD*) found differentially expressed in RNA-sequencing analysis of endometrium samples was also interrogated. The selection of these genes as interesting targets was based on literature searching (Wedel and Lömsziegler-Heitbrock, 1995; Blom et al., 2004; Hannan et al., 2004; Tapia et al., 2008; Hayashi et al., 2017; Neupane et al., 2017; Pinto De Melo et al., 2017; Tríbulo et al., 2018) and GO term analysis.

#### Quantitative Real-Time PCR Analysis

All primers were designed using Primer Blast software^3^ (Supplementary Table 2). Briefly, RT-qPCR assays were performed per duplicate in a total volume of 20 μL, containing 10 μL FastStart Universal SYBR Green Master (Roche Diagnostics Ltd., West Sussex, United Kingdom), 1.2 μL forward and reverse primer mix (300 nM final concentration), 5.6 μL nuclease-free water and 2 μL cDNA template on the ABI Prism 7500 Real-Time PCR System (Life Technologies). A total of 40 cycles were performed with the following thermo-cycling conditions for each cycle: 50°C for 2 min, 95°C for 10 min followed by 95°C for 15 s, 60°C for 1 min, 95°C for 30 s and 60°C for 15 s. The melt curve was also included to ensure specificity of amplification. The specificity of all targets was confirmed by the presence of a single sharp peak in the melt curve. A total of eight potential reference genes [Glyceraldehyde 3-Phosphate Dehydrogenase (*GAPDH*), Actin Cytoplasmic 1 (*ACTB*), 60S Ribosomal Protein L18 (*RPL18*), Peptidyl-Prolyl *Cis-Trans* Isomerase A (*PPIA*), 14-3-3 Protein Zeta/Delta (*YWHAZ*), RING Finger Protein 11 (*RNF11*), Histone H3.3 (*H3F3A*), Succinate Dehydrogenase Complex Subunit A Flavoprotein Variant (*SDHA*)] were analyzed using the geNorm function with the qbase + package (Biogazelle, Zwijnaarde, Belgium) to identify the best reference genes. Due to the high variability between samples, a total of four reference genes were selected: *RNF11, H3F3A, YWHAZ*, and *GADPH*, which were the most stably expressed (average geNorm *M* ≤ 0.5).

Primer efficiency was carried out for the genes of interest, and RT-qPCR of 1:4 dilutions of a cDNA mix from a representative pool of samples were analyzed. The presence of a single sharp peak in the melt curve as well as the standard curve was used to confirm primer specificity. The threshold cycle (Ct) for each sample was automatically calculated using the default settings within the SDS software (SDS 1.4, ABI). In order to obtain the relative expression values of the genes of interest, 2^–ΔΔCT^ method was used (Livak and Schmittgen, 2001). For each individual, the expression of the genes of interest was firstly normalized to the average of housekeeping genes previously selected (*RNF11, H3F3A, YWHAZ*, and *GADPH*) with the following formula: *Δ*Ct = Ct_gene of interest_ – Ct_(RNF11__+__H3F3A__+__YWHAZ__+__GADPH)/4_. The values of ΔΔCt were calculated normalizing the results to the mean across all individuals, including both tissue regions (vagina and ampulla), per each gene of interest. The subsequent statistical analysis was performed using ΔCt values whereas the results are represented as 2^–ΔΔCT^.

Results expressed as ΔCT were analyzed with IBM SPSS 25.0 for Windows (Armonk; New York, NY, United States). Data were checked for normal distribution (Shapiro–Wilk test) and homoscedasticity (Levene test) to confirm that parametric assumptions were fulfilled. When these premises were not, data (x) were linearly transformed using the square root (√x) and arcsine of the square root (arcsin √x). Thereafter, data (transformed or not depending on the case) were analyzed by a two-way ANOVA followed by a Sidak *post hoc* test for pair-wise comparisons. The expression of five genes (*CX3CL1*, *PLA2G10*, *TNFA*, *IL6*, and *CXCL8*), even after linear transformation, did not match parametric assumptions. For this reason, Scheirer–Ray–Hare and Mann–Whitney tests were used as non-parametric alternatives. In all cases, the significance level was established at *P* ≤ 0.05.

## Results

### Ovary Status

At the time of sample collection (24 ± 6 h after mating), a total of 13 heifers had a freshly ovulated follicle on their ovary, while the remaining animals exhibited a pre-ovulatory follicle (see Supplementary Table 1 and Supplementary Figure 1). In the control group, three animals had ovulated and four exhibited a pre-ovulatory follicle. Regarding the heifers mated with vasectomized bulls, fresh ovulation was found in six animals and only two presented a pre-ovulatory follicle. While in the intact group, four ovulations and three preovulatory follicles were observed. The proportion of animals that had a fresh ovulation was balanced across treatments for subsequent gene expression analysis.

### Effects of Seminal Plasma on the Endometrial Transcriptome

Sequencing of endometrial samples of heifers recovered 24 h after mating to intact bulls revealed a total of 22 differentially expressed genes (DEGs) compared with contemporary unmated animals (Table 1 and Supplementary File 1). Of those DEGs, 12 were up-regulated and 10 down-regulated [False discovery rate (FDR) < 0.05]. Some of the genes that exhibited the lowest expression (logFC < -2) were serine protease 2 (*PRSS2*), complement C9 (*C9*), oxytocin/neurophysin I prepropeptide (*OXT*), a novel gene encoding for carbonic anhydrase 1 (ENSBTAG00000036116) and an uncharacterized novel gene (ENSBTAG00000050072). On the other hand, the genes with greater transcript abundance (logFC > 2) levels were coiled-coil domain containing 196 (*CCDC196*), solute carrier family 24 member 2 (*SLC24A2*), UDP glucuronosyltransferase family 2-member A1 complex locus (*UGT2A1*) and interferon gamma inducible protein 47 (*IFI47*). In contrast, the endometrium of heifers exposed only to SP (by mating with a vasectomized bull) did not exhibit DEGs compared with the control group. Comparison of endometrial transcriptomes of intact and vasectomized groups revealed a total of 24 DEGs, 18 up-regulated and 6 down-regulated (Table 2 and Supplementary File 1) (FDR < 0.05). Amongst these, MHC, class II, DQ beta (*BOLA-DQB*), GSG1 like (*GSG1L*), potassium voltage-gated channel subfamily E regulatory subunit 1 (*KCNE1*) and the novel gene previously mentioned (ENSBTAG00000050072) were those that displayed lower logFC values (logFC < −2). In contrast, higher levels of expression (logFC > 2) were exhibited by interleukin 17F (*IL17F*), complement component 4 binding protein alpha (*C4BPA*), the aforementioned *IFI47* and *UGT2A1*, and a novel gene (ENSBTAG00000052851) which has been predicted to code for a protein containing an Ig-like domain.

**TABLE 1:** List of differentially expressed genes (FDR < 0.05) in endometrial samples of heifers mated with intact bulls compared with unmated heifers.

Ensembl acc. number	Gene name	Gene description	logFC
ENSBTAG00000014234	*CCDC196*	Coiled-coil domain containing 196	8.63
ENSBTAG00000043972	*SLC24A2*	Solute carrier family 24 member 2	4.15
ENSBTAG00000004040	*UGT2A1*	UDP glucuronosyltransferase family 2 member A1 complex locus	3.11
ENSBTAG00000003529	*ASAH2*	*N*-acylsphingosine amidohydrolase 2	2.82
ENSBTAG00000015727	*IFI47*	Interferon gamma inducible protein 47	2.62
ENSBTAG00000049426	*STARD2*	Phosphatidylcholine transfer protein	1.72
ENSBTAG00000037929	*ADAM28*	ADAM-like, decysin 1	1.42
ENSBTAG00000019636	*SCARA5*	Scavenger receptor class A member 5	1.06
ENSBTAG00000017722	*F5*	Coagulation factor V	0.99
ENSBTAG00000019625	*EHHADH*	Enoyl-CoA hydratase and 3-hydroxyacyl CoA dehydrogenase	0.83
ENSBTAG00000008735	*VASH1*	Vasoinhibin 1	0.77
ENSBTAG00000001728	*IGSF10*	Immunoglobulin superfamily member 10	0.72
ENSBTAG00000046307	*CEBPD*	CCAAT enhancer binding protein delta	−0.86
ENSBTAG00000011079	*C18H19orf48*	Chromosome 18 C19orf48 homolog	−0.92
ENSBTAG00000007101	*F3*	Coagulation factor III, tissue factor	−1.04
ENSBTAG00000051812	*CA1L*	Carbonic anhydrase 1-like	−2.45
ENSBTAG00000008026	*OXT*	Oxytocin/neurophysin I prepropeptide	−2.76
ENSBTAG00000039446	*PI3L*	Elafin-like	−2.78
ENSBTAG00000036116	*CA1*	Carbonic anhydrase 1	−3.39
ENSBTAG00000016149	*C9*	Complement C9	−4.08
ENSBTAG00000050072		Novel gene	−4.99
ENSBTAG00000021565	*PRSS2*	Serine protease 2	−6.05

*FDR, false discovery rate; logFC, logarithm of fold change.*

**TABLE 2:** List of differentially expressed genes (FDR < 0.05) in endometrial samples of heifers mated with intact bulls compared with heifers mated with vasectomized bulls.

Ensembl acc. number	Gene name	Gene description	logFC
ENSBTAG00000052851		Novel gene	4.57
ENSBTAG00000016835	*IL17F*	Interleukin 17F	4.50
ENSBTAG00000032884	*TNP2*	Transition protein 2	3.78
ENSBTAG00000019132	*DMP1*	Dentin matrix acidic phosphoprotein 1	3.34
ENSBTAG00000015727	*IFI47*	Interferon gamma inducible protein 47	3.18
ENSBTAG00000009876	*C4BPA*	Complement component 4 binding protein alpha	2.78
ENSBTAG00000004040	*UGT2A1*	UDP glucuronosyltransferase family 2 member A1 complex locus	2.47
ENSBTAG00000037539		Vascular cell adhesion molecule 1-like	1.81
ENSBTAG00000021764	*GLRB*	Glycine receptor beta	1.62
ENSBTAG00000002214	*TAT*	Tyrosine aminotransferase	1.38
ENSBTAG00000026779	*LYZ*	Lysozyme	1.32
ENSBTAG00000000601	*COL11A2*	Collagen type XI alpha 2 chain	1.14
ENSBTAG00000019588	*BLA-DQB*	MHC class II antigen	1.11
ENSBTAG00000034338	*C15H11orf88*	Chromosome 15 C11orf88 homolog	1.10
ENSBTAG00000021526	*RPRM*	Reprimo, TP53 dependent G2 arrest mediator homolog	1.05
ENSBTAG00000033429	*FAM229B*	Family with sequence similarity 229 member B	0.94
ENSBTAG00000024869	*CX3CL1*	C-X3-C motif chemokine ligand 1	0.78
ENSBTAG00000021522	*PLA2G10*	Group 10 secretory phospholipase A2	0.72
ENSBTAG00000012703	*GLO1*	Glyoxalase I	−0.49
ENSBTAG00000008147	*MICAL1*	Microtubule associated monooxygenase, calponin and LIM domain containing 1	−0.50
ENSBTAG00000001150	*KCNE1*	Potassium voltage-gated channel subfamily E regulatory subunit 1	−2.51
ENSBTAG00000004607	*GSG1L*	GSG1 like	−4.61
ENSBTAG00000050072		Novel gene	−4.94
ENSBTAG00000021077	*BOLA-DQB*	Major histocompatibility complex, class II, DQ beta	−5.05

*FDR, false discovery rate; logFC: logarithm of fold change.*

Three DEGs (*UGT2A1*, *IFI47* and the novel gene ENSBTAG00000050072) were found to be common of DEGs detected between the intact group and the control and those detected when comparing the intact and vasectomized groups.

### Gene Ontology (GO) Terms of DEGs

**FIGURE 2:**
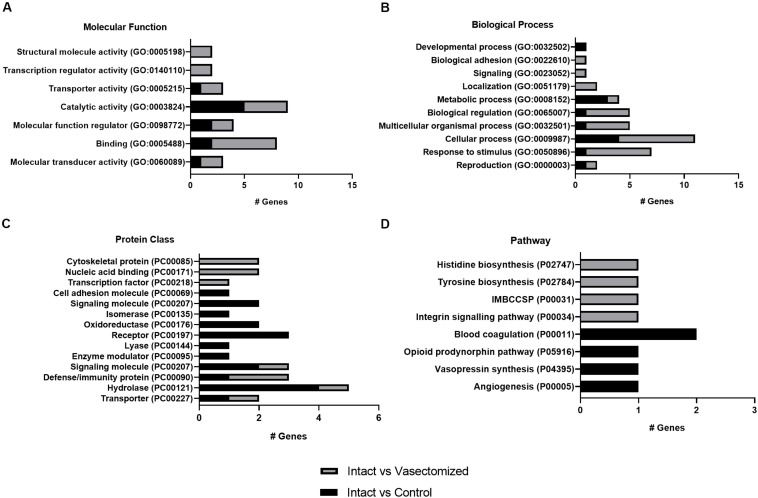
**(A–D)** Representation of GO terms for annotated DEGs in each comparison. The main categories: **(A)** molecular function, **(B)** biological process, **(C)** protein class, and **(D)** pathway were represented. IMBCCSP, inflammation mediated by chemokine and cytokine signaling pathway.

For the annotated genes in each comparison, the GO terms are shown in Figure 2. For the molecular function category, the main represented GO term was “catalytic activity” in the intact group compared with control or vasectomized samples (Figure 2A). “Cellular process” and “metabolic process” were the most represented terms for the biological process category in intact samples compared with the control (Figure 2B). Compared with the vasectomized group in the same category, in addition to “cellular process,” “response to stimulus” was the most represented term (Figure 2B). In regard to pathway category, comparing the intact group with the control, all the terms represented were related to vascular regulation (Figure 2D). In contrast, compared with the vasectomized bull treatment group, the represented terms referred to immunity modulation and amino acid biosynthesis (Figure 2D). “Hydrolase” and “receptor” were the most represented protein class terms among the differentially regulated genes in the endometrium exposed to sperm and SP compared with the control samples (Figure 2C). On the other hand, compared with vasectomized samples, the most represented protein class terms were “cytoskeletal protein” and “immunity protein” (Figure 2C).

### Effects of Seminal Plasma on Gene Expression in the Vagina and Oviduct

Based on the results obtained from the endometrial RNA-sequencing, we were interested in studying whether gene expression changes are more dramatic at the site of semen deposition (the vagina), and whether these changes can propagate to more distal regions (the oviductal ampulla). Thus, six DEGs from the sequencing analysis were selected (*PLA2G10*, *CX3CL1*, *C4BPA*, *PRSS2*, *BLA-DQB*, and *CEBPD*) based on evidence of their reproductive function found in the literature (Wedel and Lömsziegler-Heitbrock, 1995; Blom et al., 2004; Hannan et al., 2004; Tapia et al., 2008; Hayashi et al., 2017; Neupane et al., 2017; Pinto De Melo et al., 2017; Tríbulo et al., 2018), as well as their GO terms. In addition to these, *IL6*, *IL1A*, *TNFA*, and *IL8* expression was also assessed, as these are genes that have been observed to be regulated by SP in several species (O’Leary et al., 2004; Sharkey et al., 2012b; Schjenken et al., 2015; Introini et al., 2017; Ibrahim et al., 2019).

**FIGURE 3:**
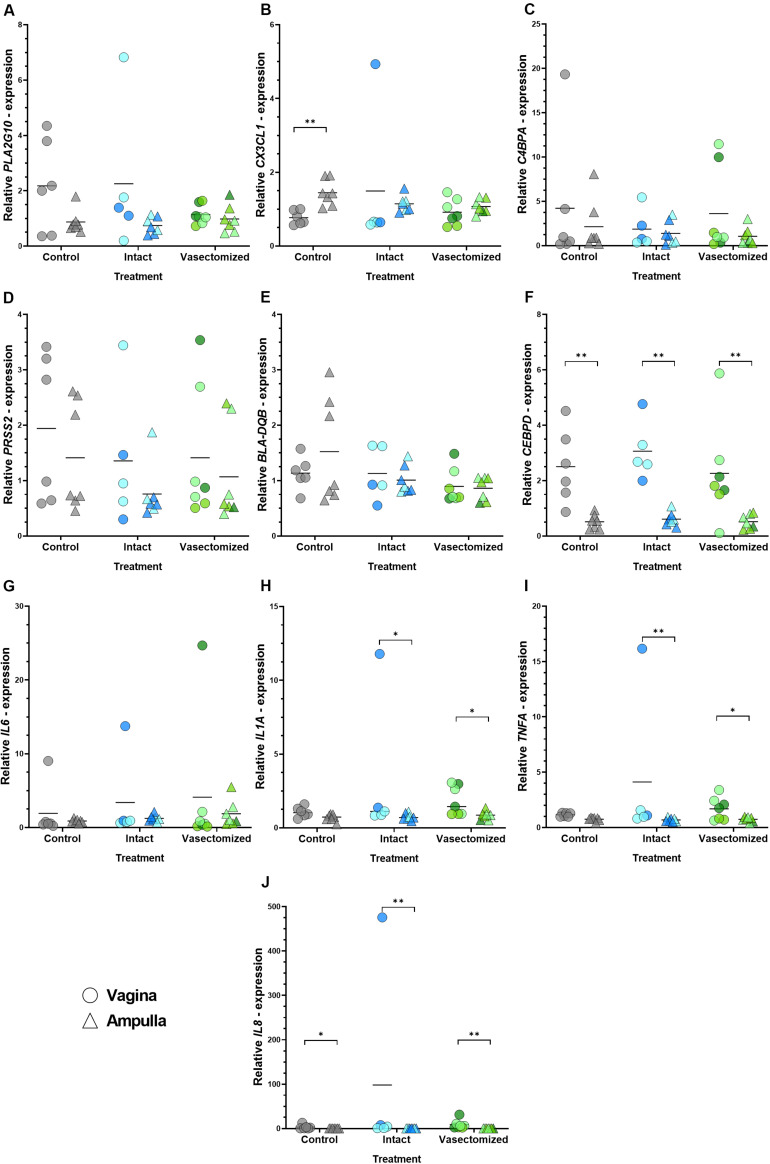
**(A–J)** Relative expression values of all the genes assessed: **(A)** *PLA2G10*, **(B)** *CX3CL1*, **(C)** *C4BPA*, **(D)** *PRSS2*, **(E)** *BLA-DQB*, **(F)** *CEBPD*, **(G)** *IL6*, **(H)** *IL1A*, **(I)** *TNFA*, **(J)** *IL8* in the vagina (circles) and oviductal ampulla (triangle) in the different experimental groups (control, intact or vasectomized). Each circle/triangle refers to an individual heifer. Different color shades within groups correspond to the bull that was mated to that particular heifer, blue for intact group (*n* = 2 bulls) and green for vasectomized group (*n* = 3). Bars represent the mean of relative expression values and asterisks indicate significant (^∗^*P* < 0.05, ^∗∗^*P* < 0.01) differences.

Differences in relative expression of *CEBPD* (*P* < 0.01; Figure 3F) and *IL8* (*P* < 0.05 in the control group and *P* < 0.01 in the intact and vasectomized groups; Figure 3J) between regions were observed in all groups, being up-regulated in the vagina in comparison with the ampulla, whereas *CX3CL1* was down-regulated in the vagina compared with the ampulla, only in the control group (*P* < 0.01; Figure 3B). In addition, *TNFA* (*P* < 0.05 in the intact group and *P* < 0.01 in the vasectomized group; Figure 3I) and *IL1A* (*P* < 0.05; Figure 3H) were up-regulated in the vagina compared with the ampulla in heifers that had been mated to an intact or a vasectomized bull, but not in unmated heifers. The remaining genes did not exhibit region-specific changes (Figures 3A,C–E,G). Conversely, when relative abundance of these genes was compared between treatment groups, no differences were detected (*P* > 0.05). It is also important to note that for many genes, especially those related to inflammation, there was considerable variability between animals.

## Discussion

The main findings of this study are: (1) mating to an intact bull induces subtle changes in the endometrial transcriptome; however, (2) these transcriptomic changes are not observed in heifers mated to vasectomized bulls; (3) expression of *PLA2G10*, *CX3CL1*, *C4BPA*, *PRSS2*, *BLA-DQB*, *CEBPD*, *IL1A*, *IL6*, *TNFA*, and *IL8* in the vagina and ampulla did not differ between treatments; and (4) *TNFA* and *IL1A* exhibited regional differences between vagina and ampulla of heifers mated to intact or vasectomized bulls.

Seminal plasma is a complex fluid the composition of which is determined by the size, storage capacity, and secretory output of different organs of the male reproductive tract, which in the bull include: testes, epididymides, ampullae of the vasa deferentia, seminal vesicles, prostate and bulbourethral glands. After completion of spermatogenesis, sperm enter the epididymis bathed in fluid produced by the rete testis, which will be absorbed in its majority by the epididymal epithelium (Amann et al., 1974). However, secreted factors of epididymal and/or testicular origin are found in the ejaculate. This is evidenced by studies comparing ejaculates before and after vasectomy, which show lower concentration of amino acids in bulls (Alexander et al., 1971), and proteins in humans (Batruch et al., 2011), after the procedure. Despite this, vasectomy does not lead to a decrease in bull ejaculate volume (Alexander et al., 1971), indicating a more prominent role of the male accessory glands in the production of SP components. It is challenging to precisely calculate the contribution of each gland to the final fluid volume and composition (Seidel and Foote, 1970); however, vesiculectomy (excision of the seminal vesicles) in the bull leads to a more than 50% decrease in ejaculate volume, together with a reduction in total protein and ion concentrations (chloride, potassium, calcium, sodium), lower sperm motility, viability and morphology, and increased pH (Faulkner et al., 1968; Alexander et al., 1971).

Growing evidence exists for a role of SP in the modulation of cellular and molecular events in the maternal tract of several species during early pregnancy (Gangnuss et al., 2004; O’Leary et al., 2004, 2006; Bromfeld, 2016; Song et al., 2016; Glynn et al., 2017; Morgan and Watkins, 2020). However, most of the *in vivo* evidence comes from pigs and mice, species in which SP reaches the uterus (Gangnuss et al., 2004; O’Leary et al., 2004, 2006; Bromfield et al., 2014; Song et al., 2016; Glynn et al., 2017). Because of the relatively low volume of ejaculate in men (an average of 3.7 mL, Cooper et al., 2009) and bulls (around 5 mL), and the fact that the ejaculate is deposited in the vagina, it is questionable whether any SP reaches the uterus. Therefore, it is not clear whether this fluid has a critical role in the modulation of the uterine environment in these species. At the time of ejaculation, however, sperm come into contact with SP, leading to proteins binding tightly to the sperm plasma membrane (Pini et al., 2016). For example, seminal vesicle-derived Binder of Sperm Proteins (BSP) −1, −3, and −5 (previously called PDC-109 or BSP-A1/A2, BSP-A3 and BSP-30 kDa respectively), which make up approximately 50% of total protein in SP (Nauc and Manjunath, 2000), bind to sperm and play important roles during capacitation (Manjunath and Thérien, 2002) and formation of the sperm oviductal reservoir (Gwathmey et al., 2003, 2006). Thus, it is possible that in intravaginal ejaculators, sperm can act as a vehicle of SP proteins that interact with the reproductive epithelium to induce changes in the maternal environment. Indeed, bull sperm has been shown to interact with endometrial cells and induce a proinflammatory response *in vitro* (Elweza et al., 2018; Ezz et al., 2019).

To address this lack of basic knowledge, this study aimed to determine the effects of bovine SP and sperm exposure during natural mating on the endometrial transcriptome. Additionally, gene expression changes were assessed at the site of semen deposition (vagina) and the distal region where gamete interaction takes place (the oviductal ampulla) to determine whether SP-induced changes can propagate throughout the female reproductive tract.

In the present study, heifers were mated between 0 and 6 h after seen in standing estrus. Average time from estrus onset to ovulation is 27 h (Walker et al., 1996; Valenza et al., 2012; Randi et al., 2018). Thus, some animals had ovulated by the time of sample collection (24 ± 6 h after mating) whereas the rest exhibited a pre-ovulatory follicle. Ovulation and estrous cycle are orchestrated by an accurate hormonal regulation, and under this regulation, the endometrium experiments functional and morphological changes (Arai et al., 2013). In order to avoid any possible confounding factors due to ovulation having occurred or not, the number of ovulated and non-ovulated heifers that were analyzed by RNA-sequencing and RT-qPCR was balanced between treatments.

Strikingly, when heifers were exposed to SP in the absence of sperm and testicular and epididymal secretions (i.e., mated to a vasectomized bull) the endometrial RNA-sequencing analysis did not reveal any DEGs in comparison with samples from unmated animals. Conversely, the endometrial transcriptome of heifers mated to intact bulls differed from the control and vasectomized groups, exhibiting differential regulation of a small number of genes that may play a role in bovine fertility. Although, these results could be due to SP reaching the uterus in both treatments, but only testicular of epididymal factors inducing a response; it is more likely that, in cattle, SP does not reach the uterus in the 24 h following natural mating, at least in the absence of a vehicle, such as sperm.

When compared with control endometrial samples, endometrium obtained from heifers mated to an intact bull exhibited up-regulation of 12 genes and down-regulation of 10 genes. Amongst these genes regulated by mating, some have been shown to participate in tissue remodeling, an important step preparing endometrium to embryo implantation. The gene coding for scavenger receptor class A, member 5 (*SCARA5)*, which participates in innate immunity (Jiang et al., 2006) was up-regulated. This gene has also been observed to be up-regulated in the endometrium of cows at day 20 of pregnancy (Mansouri-Attia et al., 2009), and has been proposed to play a role in the regulation of histotroph secretion and tissue remodeling, two critical processes for embryo implantation (Vitorino Carvalho et al., 2019). Additionally, serine protease 2 (*PRSS2*), which also participates in tissue remodeling by type 1 collagen degradation, was down-regulated by exposure to SP and sperm at mating. Interestingly, this gene is up-regulated in the endometrium of repeat breeder cows, those that are cycling normally and without clinical abnormalities but that fail to conceive after at least two successive inseminations (Hayashi et al., 2017). Mating to an intact bull also affected genes involved in cell proliferation, such as CCAAT enhancer binding protein delta (*CEBPD*), which was found to be down-regulated in the endometrium of heifers mated with intact bulls. This gene belongs to the C/EBP leucine-zipper transcription factor family involved in fat and hematopoietic progenitor cells differentiation (Wedel and Lömsziegler-Heitbrock, 1995). Another member of this family, CEBPB, has been identified as a regulator of proliferative events during decidualization in mice (Mantena et al., 2006). In regard to modulation of innate immunity, the gene coding for component 9 of complement system (*C9*) was down-regulated in the endometrium of heifers mated with intact bulls. The C9 component participates in the final steps of the complement cascade, in the formation of membrane attack complex (MAC), which mediates the formation of channels in the target cell membrane, leading to cell lysis and death (Janeway et al., 2001). Despite its importance, the function of the complement system in the context of reproduction is not well known. For instance, complement regulatory proteins have been found in bull sperm surface, such as CD59 (Byrne et al., 2012), which prevents the formation of MAC (Janeway et al., 2001). These complement regulatory proteins were also identified in human and mouse sperm and they have been proposed to play a role protecting sperm in the female tract (Harris et al., 2006). Moreover, mating with an intact bull resulted in a down-regulation of endometrial oxytocin (*OXT*). In cattle, high levels of OXT have been reported to impair embryo survival by promoting uterine secretion of prostaglandin *F*_2α_ (PGF_2α_), which induces luteolysis and consequently a drop in progesterone (Lemaster et al., 1999). Although endometrial OXT production has been observed also in mares (Bae and Watson, 2003), it is not known the locally effect of the OXT secreted by the endometrium during early pregnancy in cattle.

When comparing endometrial samples from heifers mated to intact bulls to those from heifers mated to vasectomized bulls, 18 genes were found to be up-regulated and four down-regulated. Amongst the up-regulated genes, literature on *CX3CL1*, *VCAM1-like*, *C4BPA*, *PLA2G10*, *IFI47*, *IL17F* and *BLA-DQB* suggest different roles of these genes in early pregnancy in different species. The chemokine CX3CL1 (C-X3-C motif chemokine ligand 1) has been identified as a potential bovine embryokine (Tríbulo et al., 2018). In addition, CX3CL1 induces recruitment of leukocytes during early pregnancy (Hannan et al., 2004), and also promotes trophoblast migration in women (Hannan et al., 2006). With importance for implantation, VCAM1 has been shown to be involved in the adhesion of the bovine conceptus to the endometrium (Bai et al., 2014). Further, in women with unexplained infertility the endometrial expression of *VCAM1* at the peri-implantation stage was significantly lower than control women (Konac et al., 2009). Additionally, the mRNA levels of complement component 4-binding protein alpha (*C4BPA*), a key inhibitor of the complement system (Blom et al., 2004), were increased during the implantation window in women (Tapia et al., 2011), but decreased in women which suffered repeated implantation failure and unexplained recurrent spontaneous abortion (Lee et al., 2007; Tapia et al., 2008). Although cattle and human implantation differs significantly, in both the time at which it takes place (around day 9 in humans and starting at day 21 in cattle) and the structure of the placenta (hemochorial in humans and epitheliochorial in bovine), it is likely that the up-regulation of *C4BPA* and *VCAM1* induced by exposure to SP and sperm during mating regulates peri-implantation events in cattle. On the other hand, group 10 secretory phospholipase A2 (*PLA2G10*), which was up-regulated in intact group samples, belongs to phospholipase A2 enzyme family, which is known to participate in inflammatory processes and to catalyze the release of arachidonic acid from phospholipids, needed to prostaglandin production (reviewed in Capper and Marshall, 2001). In addition, this gene has been associated with fertility in beef cattle (Neupane et al., 2017), and it was found highly down-regulated in the uterus of cows with negative energy balance (Wathes et al., 2009) which typically have lower pregnancy rates.

From an immunological point of view, the expression of interferon gamma inducible protein 47 (*IFI47*) was also found to be up-regulated in the endometrium of heifers mated with intact bulls in comparison with those mated to vasectomized bulls. Although its function in the uterus has not been defined, *IFI47* mRNA was more abundant in the endometrium of high fertility heifers compared to heifers classified as infertile (Minten et al., 2013). In addition, *IFI47* was up-regulated in the endometrium of heifers 13 days after embryo transfer (Spencer et al., 2013), probably under stimulation of interferon-tau secreted by the conceptus. Another immune-related gene up-regulated by mating with intact bulls is interleukin 17 F (*IL17F*), a cytokine produced by T helper 17 lymphocytes (reviewed in Iwakura et al., 2011). A similar response in expression of *IL17A* was observed by Ibrahim et al. (2019). Both IL17A and IL17F share biological functions; indeed, both are highly homologous, can bind to the same receptor and moreover, can be secreted as heterodimers or homodimers (reviewed in Iwakura et al., 2011). Ibrahim et al. (2019) observed increased levels of *IL17A* after exposing endometrial cell cultures to SP and sperm or sperm alone, but not SP in the absence of sperm. Also, up-regulation of *IL17A* took place in *vivo*, after uterine infusion of semen (a combination of SP and sperm) but not SP alone (Ibrahim et al., 2019). Together with the data obtained in the present study, this suggests that regulation of *IL17* expression in the bovine endometrium is mediated by sperm action, not SP. This is interesting to note as, in mice, mating to a vasectomized male induces a similar increase in endometrial *IL17A* expression than mating with an intact male (Song et al., 2016). This gives weight to our hypothesis that in species that ejaculate intravaginally, sperm play a more important role than SP in regulation of the female reproductive environment. Moreover, the endometrial up-regulation of *IL17A* in bovine explants has been shown to be exclusively regulated by elongated Day 15 conceptuses, but not by interferon-tau, which is the main signal of pregnancy recognition in cattle, suggesting a role in the embryo – endometrium crosstalk during early pregnancy in cattle (Sánchez et al., 2019). During pregnancy, the maternal immune system must tolerate the presence of an embryo that expresses paternal antigens. The increase in endometrial *IL17* expression observed after mating in the aforementioned studies is reflective of an increase in the population of T helper 17 lymphocytes (Song et al., 2016), which probably participate in the establishment of this maternal tolerance toward paternal antigens. Another mechanism of maternal tolerance driven by male factors seems to be the regulation of the MHC. In the present study, mating to an intact bull elicited down-regulation of Bovine Lymphocyte antigen (referred to as MHC in other species) class II, DQ beta (*BOLA-DQB*), consistent with its down-regulation in pregnant heifers after natural breeding (Dickinson et al., 2018). Conversely, another MHC class II member, *BLA-DQB* was up-regulated in endometrial samples of heifers mated with intact bulls. Indeed, a list of genes of MHC class II family members, including *BLA-DQB* and *BOLA-DQB*, have been associated with reproductive performance in cattle (Pinto De Melo et al., 2017).

The lack of a response to mating with a vasectomized bull, together with the discovery of genes that were regulated by mating to intact bulls, led to the analysis of tissues that have direct contact with seminal fluid (i.e., the vagina). The ejaculation site during natural conception is an important factor to take into consideration since a recent study has shown that SP infusion into the vagina, but not into the uterus, could influence the levels of endometrial epidermal growth factor (Badrakh et al., 2020), which has been associated with fertility restoration in repeat breeder cows (Katagiri and Takahashi, 2006). In this sense, we were also interested in studying how far into the reproductive tract these changes could be observed, so gene expression analysis in the oviductal ampulla was also undertaken. Based on the sequencing results, genes identified as possible key regulators of uterine environment and pregnancy success (*PLA2G10*, *CX3CL1*, *C4BPA*, *PRSS2*, *BLA-DQB*, and *CEBPD*) were selected. In addition, the expression of inflammatory mediators regulated by SP components in other species (*IL1A*, *IL6*, *TNFA*, and *IL8*; O’Leary et al., 2004; Sharkey et al., 2012b; Schjenken et al., 2015; Introini et al., 2017; Ibrahim et al., 2019) was also analyzed. However, no difference between treatments was observed in the expression of any gene in the vagina or the oviduct. These results are not consistent with data in other species: in the mouse, *Il6* was reduced in the oviduct of females mated to males that had undergone a vesiculectomy with or without a vasectomy (Bromfield et al., 2014); while in the pig, natural mating induces up-regulation of *CEBPD* in the ampulla (Alvarez-Rodriguez et al., 2019); finally, in human cervix, an increase in *IL1A*, *IL6* and *IL8* is observed after coitus (Sharkey et al., 2012b). It is important to highlight, however, that the heifers in the present study had been estrous synchronized (both mated and control animals) with an intravaginal device that was removed 48 h prior to sample collection. Although all the animals were managed under the same conditions, the resulting manipulation could have had an impact on the inflammatory status of the vagina. Indeed, a higher dispersion within group is observed in the expression of inflammatory genes in the vagina, than in the ampulla region. This is especially noticeable in the intact group, were one heifer exhibits very high expression of *CX3CL1, IL6, IL1A, TNFA*, and *IL8*. Despite the lack of treatment effect, some genes were shown to be differentially expressed between tissues. A higher expression of *CX3CL1* was found in the ampulla compared with the vagina in control samples. In women, CXCL3 is present throughout the oviduct and, interestingly, its receptor was found in ejaculated sperm (Zhang et al., 2004). Conversely, *CEBPD* and *IL8* were found up-regulated in the vagina samples compared with ampulla in all treatments. On the one hand, the expression of *CEBPD* is a crucial factor during inflammatory acute-phase response, under regulation of a range of cytokines and other inflammatory agents (reviewed in Ramji and Foka, 2002) and, on the other hand, IL8 is well known to be a potent neutrophil chemoattractant (Leonard and Yoshimura, 1990). Therefore, a higher basal expression of both genes in the vagina compared with the ampulla might be expected since this tissue has contact with the outside and, thus, is more prone to environmental/external contaminants.

Interestingly, *IL1A* and *TNFA* were more highly expressed in vagina than in the ampulla tissue of heifers mated either to intact or vasectomized bulls, while control heifers did not exhibit this region-specific difference, suggesting a modulatory role induced by mating. In human exposure of ectocervical explants to SP resulted in the increase of *IL1A* and *TNFA* expression levels (Introini et al., 2017). Further, unprotected vaginal coitus, but not condom protected, induced the expression of *IL1A* in women (Sharkey et al., 2012b). However, in our study, the mechanical stimulus of mating cannot be ruled out.

## Conclusion

The lack of changes in the endometrial transcriptome and in the expression of selected genes in the vagina and oviduct after mating to a vasectomized male do not support a role of SP (in the absence of sperm nor testicular and epididymal secretions) in regulating early pregnancy and uterine environment in cattle. Rather, the subtle changes in the transcriptome of the endometrium and the vagina seem to be elicited by sperm. These data indicate that, in species that ejaculate intravaginally, sperm play a more critical role in the modulation of the female environment. This is most apparent when looking at regulation of *IL17*, which is driven by SP in mice (Song et al., 2016) and by sperm in cattle. However, further research is needed to elucidate the role of inherent sperm proteins or SP proteins that attach to sperm at ejaculation.

## Data Availability Statement

Gene expression data are publicly available at the Dryad Digital Repository (https://doi.org/10.5061/dryad.s7h44j14r).

## Ethics Statement

The animal study was reviewed and approved by the Animal Research Ethics Committee of University College Dublin, Ireland and licensed by the Health Products Regulatory Authority (HPRA), Ireland, in accordance with Statutory Instrument No. 543 of 2012 (under Directive 2010/63/EU on the Protection of Animals used for Scientific Purposes).

## Author Contributions

SR carried out the laboratory work, analyzed the results, and wrote the draft. YM-O contributed to the laboratory work and the analysis of results. BF-F, JS, SB-A, MM, and DK carried out the animal work, including handling of bulls and heifers, estrus detection, mating and sample collection. SB and TS performed the RNA library preparation and sequencing analysis. MY performed the statistical analysis of the data. BF-F created the illustrations for Figure 1. PL, JS, MY, and BF-F contributed to the critical revision of the manuscript. BF-F, PL, and JS designed the study. All authors read and approved the final manuscript.

## Conflict of Interest

The authors declare that the research was conducted in the absence of any commercial or financial relationships that could be construed as a potential conflict of interest.

## Footnotes

1 https://ccb.jhu.edu/software/fqtrim/

2 pantherdb.org

3 https://www.ncbi.nlm.nih.gov/tools/primer-blast/
