# Supplementary material for: Mating to Intact, but Not Vasectomized, Males Elicits Changes in the Endometrial Transcriptome: Insights From the Bovine Model
Source: Front Cell Dev Biol. 2020 Jul 10;8:547. doi: 10.3389/fcell.2020.00547 (PMC7381276; doi:10.3389/fcell.2020.00547)
Supplement: Supplementary file 2 [file Table_1.docx]

Supplementary Material

# Supplementary Data

**Supplementary Table 1:** Relation of heifers and bulls used in the study. The time passed since mating (in the case of intact or vasectomized treatments) or standing estrus (in the case of control animals) is indicated, together with the ovarian structures observed at recovery time.

| Heifer | Treatment | Bull | Time from mating/estrus to slaughter | Ovarian structures | Used in RNA-sequencing analysis |
| --- | --- | --- | --- | --- | --- |
| Ctr_1 | Control | N/A | 24 h | Fresh ovulation | ✓ |
| Ctr_2 | Control | N/A | 24 h | Fresh ovulation | ✓ |
| Ctr_3 | Control | N/A | 22 h | Preovulatory follicle | ✓ |
| Ctr_4 | Control | N/A | 23 h | Preovulatory follicle | ✓ |
| Ctr_5 | Control | N/A | 29 h | Preovulatory follicle | ✓ |
| Ctr_6 | Control | N/A | 30 h | Fresh ovulation | ✓ |
| Ctr_7 | Control | N/A | 24 h | Preovulatory follicle | - |
| Intact_1 | Intact | I1 | 30 h | Fresh ovulation | ✓ |
| Intact_2 | Intact | I2 | 24 h | Fresh ovulation | ✓ |
| Intact_3 | Intact | I1 | 28 h | Fresh ovulation | ✓ |
| Intact_4 | Intact | I2 | 29 h | Fresh ovulation | ✓ |
| Intact_5 | Intact | I1 | 29 h | Preovulatory follicle | ✓ |
| Intact_6 | Intact | I2 | 28 h | Preovulatory follicle | ✓ |
| Intact_7 | Intact | I2 | 24 h | Preovulatory follicle | - |
| Vasec_1 | Vasect | V1 | 30 h | Fresh ovulation | ✓ |
| Vasec_2 | Vasect | V2 | 21 h | Preovulatory follicle | ✓ |
| Vasec_3 | Vasect | V3 | 29 h | Fresh ovulation | ✓ |
| Vasec_4 | Vasect | V2 | 24 h | Preovulatory follicle | ✓ |
| Vasec_5 | Vasect | V2 | 23 h | Fresh ovulation | ✓ |
| Vasec_6 | Vasect | V1 | 23 h | Fresh ovulation | ✓ |
| Vasec_7 | Vasect | V1 | 23 h | Fresh ovulation | - |
| Vasec_8 | Vasect | V3 | 24 h | Fresh ovulation | - |

**Supplementary Table 2:** Gene symbol, forward and reverse primer sequences, amplicon size and accession number for each gene assessed by RT-qPCR.

| Gene Symbol | Forward Primer Sequence (5′-3′) | Reverse Primer Sequence (5′-3′) | Size (bp) | Accession Number (Bos taurus) |
| --- | --- | --- | --- | --- |
| *GAPDH* | TTCTACTGGCGCTGCCAAGG | GATCCACAACAGACACGTTGGG | 107 | NM_001034034.2 |
| *ACTB* | CAGCAGATGTGGATCAGCAAGC | AACGCAGCTAACAGTCCGCC | 91 | NM_173979.3 |
| *RPL19* | GAAAGGCAGGCATATGGGTA | TCATCCTCCTCATCCAGGTT | 86 | NM_001040516.1 |
| *PPIA* | CATACAGGTCCTGGCATCTTGTCC | CACGTGCTTGCCATCCAACC | 108 | NM_178320.2 |
| *YWHAZ* | TGAAGCCATTGCTGAACTTG | TCTCCTTGGGTATCCGATGT | 114 | NM_174814.2 |
| *RNF11* | TCCGGGAGTGTGTGATCTGTATGAT | GCAGGAGGGGCACGTGAAGG | 131 | NM_001077953.1 |
| *H3F3A* | CATGGCTCGTACAAAGCAGA | ACCAGGCCTGTAACGATGAG | 136 | NM_001014389.2 |
| *SDHA* | ACTTCACCGTTGATGGCAATAA | CGCAGAAATCGCATCTGAAA | 59 | NM_174178.2 |
| *PLA2G10* | GTGTCAAGTGTGAACCAACGG | GTTGTACTCTGCTCGGGCTA | 95 | XM_003587818.5 |
| *CX3CL1* | AATGACCCAGAAGATTCCCGAG | TTCAGGCTACATGACAGCTCC | 88 | XM_015475803.2 |
| *C4BPA* | TGGGCAAGTGATAGTTAAGACAGAT | AATGGTTGGAGGAGGTTCACAC | 181 | NM_174252.3 |
| *PRSS2* | ATCCGCCACCCCAAGTACAG | GGGTAGTTGACGCCACTGCT | 185 | NM_174690.1 |
| *BLA-DQB* | AGTACGTGCGGTTCGACAG | TAGGTTCCACTCGCCGCT | 186 | NM_001034668.3 |
| *CEBPD* | TTCAGCGCCTACATCGACTC | GTTGAAGAGGTCGGCGAAGA | 81 | NM_174267.2 |
| *IL6* | GCGCATGGTCGACAAAATCT | AAATCGCCTGATTGAACCCAGA | 158 | NM_173923.2 |
| *IL1A* | GGTCCATACCTGACGGCTACTA | CAGGCATCTCCTTTAGCAAGACG | 178 | NM_174092.1 |
| *TNFA* | TGGTTCAAACACTCAGGTCCTCT | TACGAGTCCCACCACCGGA | 106 | NM_173966.3 |
| *IL8* | TGAGGACATGTGGAAGCACTTTA | TCCCATTTCTCCAAATTCATGCAC | 73 | NM_173925.2 |

**Supplementary Figure 1:** Representative pictures of the ovaries of heifers slaughtered 24 h (±6 h) after mating. **A)** Left ovary containing a pre-ovulatory follicle; regressing corpus luteum on the right ovary. **B)** Right ovary containing a freshly ovulated follicle.


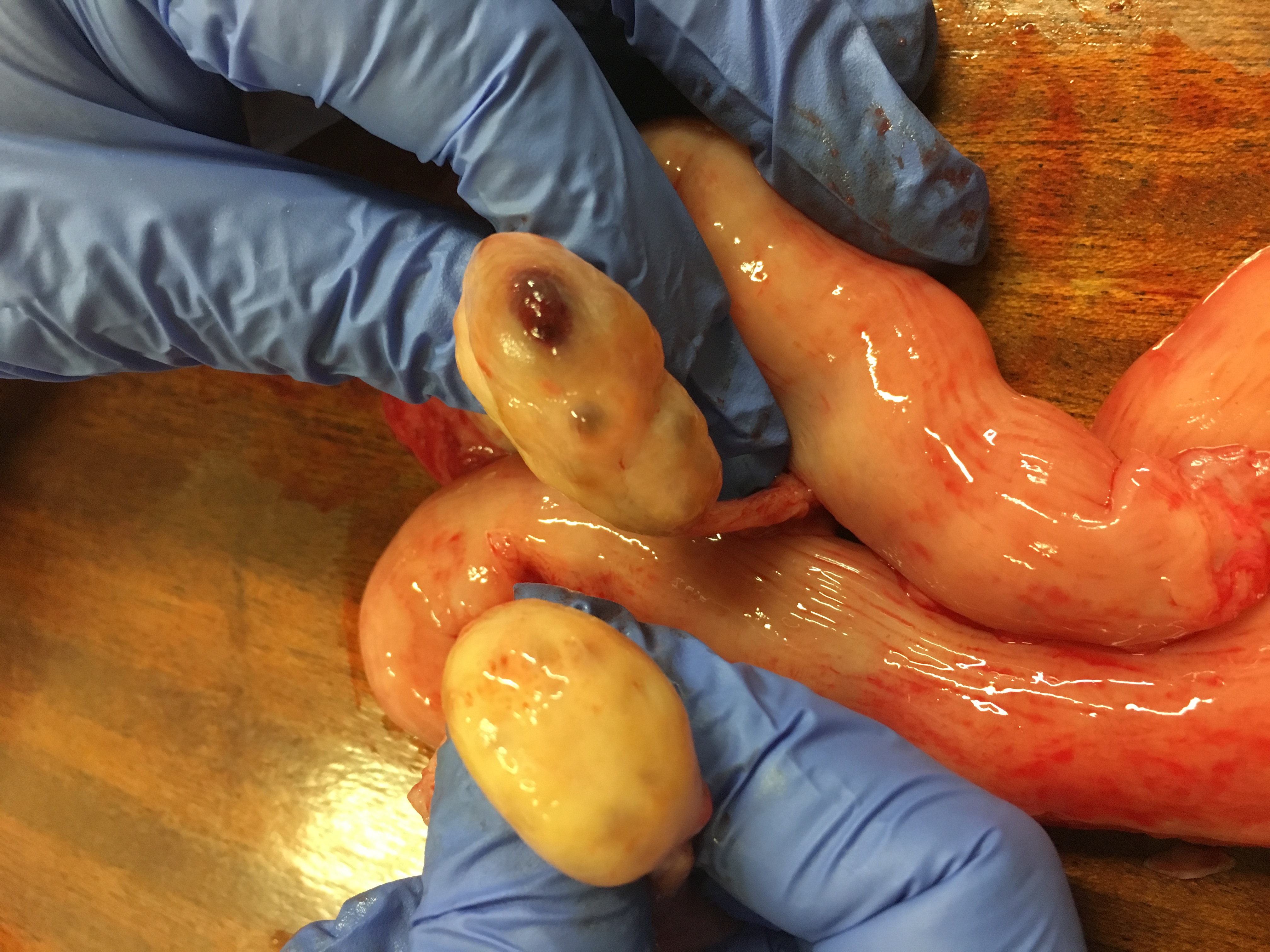

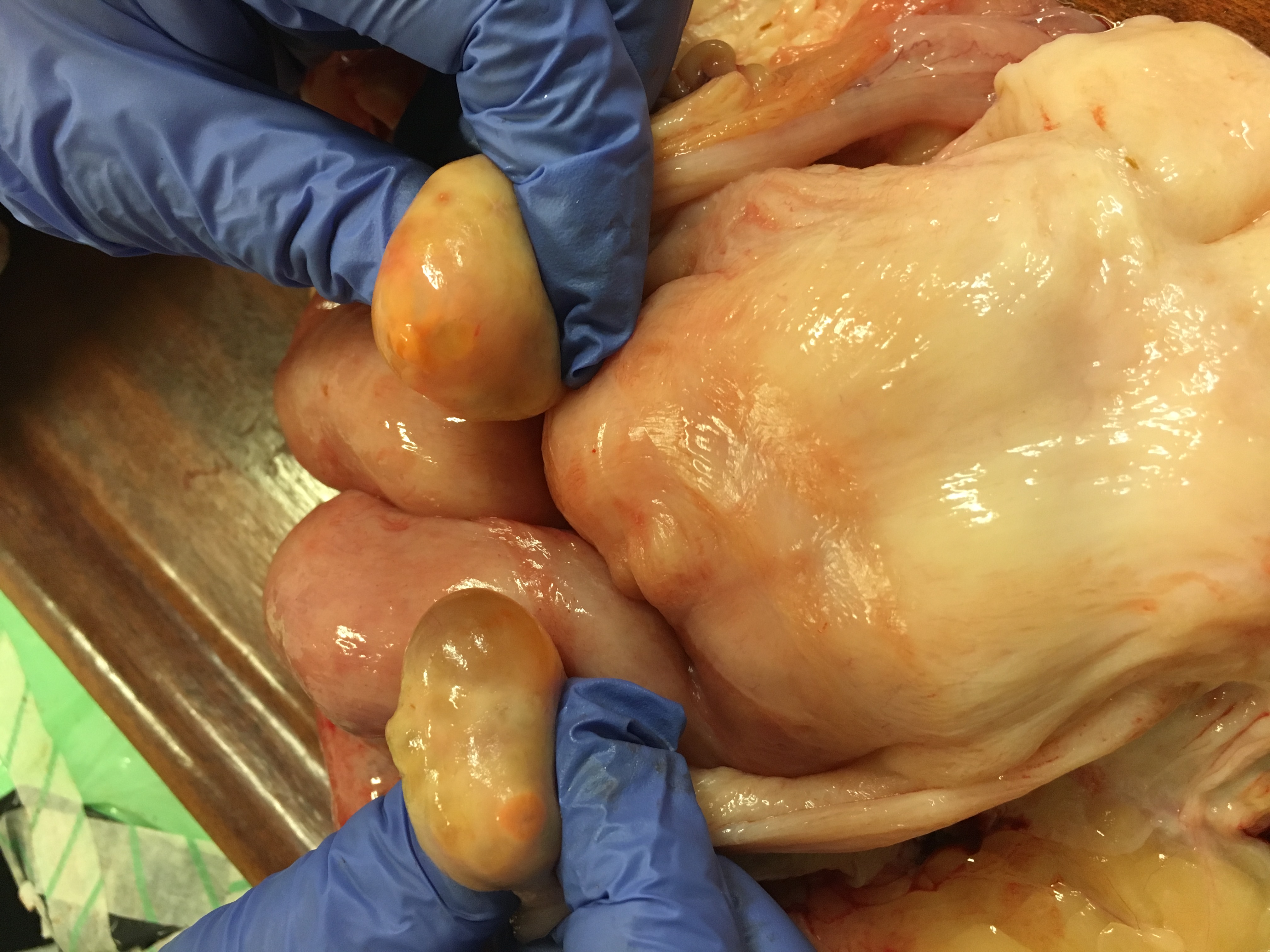


**A**

**B**
